# Maternal LPS Exposure during Pregnancy Impairs Testicular Development, Steroidogenesis and Spermatogenesis in Male Offspring

**DOI:** 10.1371/journal.pone.0106786

**Published:** 2014-09-25

**Authors:** Hua Wang, Lu-Lu Yang, Yong-Fang Hu, Bi-Wei Wang, Yin-Yin Huang, Cheng Zhang, Yuan-Hua Chen, De-Xiang Xu

**Affiliations:** 1 Department of Toxicology, School of Public Health, Anhui Medical University, Hefei, Anhui, China; 2 Anhui Provincial Key Laboratory of Population Health & Aristogenics, Hefei, Anhui, China; 3 Department of Histology and Embryology, Anhui Medical University, Hefei, Anhui, China; Qingdao Agricultural University, China

## Abstract

Lipopolysaccharide (LPS) is associated with adverse developmental outcomes including embryonic resorption, fetal death, congenital teratogenesis and fetal growth retardation. Here, we explored the effects of maternal LPS exposure during pregnancy on testicular development, steroidogenesis and spermatogenesis in male offspring. The pregnant mice were intraperitoneally injected with LPS (50 µg/kg) daily from gestational day (GD) 13 to GD 17. At fetal period, a significant decrease in body weight and abnormal Leydig cell aggregations were observed in males whose mothers were exposed to LPS during pregnancy. At postnatal day (PND) 26, anogenital distance (AGD), a sensitive index of altered androgen action, was markedly reduced in male pups whose mothers were exposed to LPS daily from GD13 to GD 17. At PND35, the weight of testes, prostates and seminal vesicles, and serum testosterone (T) level were significantly decreased in LPS-treated male pups. At adulthood, the number of sperm was significantly decreased in male offspring whose mothers were exposed to LPS on GD 13–17. Maternal LPS exposure during gestation obviously diminished the percent of seminiferous tubules in stages I–VI, increased the percent of seminiferous tubules in stages IX–XII, and caused massive sloughing of germ cells in seminiferous tubules in mouse testes. Moreover, maternal LPS exposure significantly reduced serum T level in male mice whose mothers were exposed to LPS challenge during pregnancy. Taken together, these results suggest that maternal LPS exposure during pregnancy disrupts T production. The decreased T synthesis might be associated with LPS-induced impairments for spermatogenesis in male offspring.

## Introduction

Lipopolysaccharide (LPS) is a toxic component of cell walls in Gram-negative bacteria and is widely used to establish a well-known model of bacterial infection. Humans are constantly exposed to low levels of LPS through infection, gastrointestinal distress and alcohol drinking [1], [2]. High levels of LPS have also been detected in women with bacterial vaginosis [3]. In human, Gram-negative bacterial infections are recognized as a cause of fetal loss and preterm labor [4], [5]. Mimicking maternal infection by exposing the pregnant rodents to LPS during the first trimester resulted in embryonic resorption and fetal death [6], [7]. Maternal LPS exposure during the second trimester caused fetal death and preterm delivery [8]. We and others found that maternal LPS exposure during the third trimester led to fetal death, fetal growth restriction, skeletal development retardation, and preterm labor [9]–[13]. Several studies including ours showed that maternal LPS exposure resulted in fetal teratogenesis in rats [14], mice [15], [16], and golden hamsters [17].

Recently, results from epidemiological studies and animal experiments showed that prenatal exposure to LPS could lead to structural damage and dysfunction for hippocampal neuron and cerebral cortex, thereby inducing schizophrenia, autism and cerebral palsy at adulthood [18], [19]. Our previous results also showed maternal LPS exposure during the middle or late gestation caused an age-dependent impairments of neurobehavioral development, such as spatial learning and memory ability, anxiety and exploration activity, sensorimotor and species-typical behaviors in offspring at adulthood [20], [21]. However, little is known about the effects of maternal LPS exposure during pregnancy on reproduction and endocrine function in male offspring.

In the current study, we investigated the effects of maternal mice exposed to LPS during pregnancy on testicular development, steroidogenesis and spermatogenesis in male offspring. Results showed that maternal LPS exposure during pregnancy led to a significant decrease in body weight and abnormal Leydig cell aggregations in male fetuses on GD18, a reduction in the weight of testes, prostates and seminal vesicles in male offspring at PND35, a decline of serum testosterone level in male offspring at PND35 and PND63, a decrease in sperm count and massive sloughing of germ cells in seminiferous tubules of male offspring at PND63.

## Materials and Methods

### Chemicals and reagents

Lipopolysaccharide (Escherichia coli LPS, serotype 0127:B8) was purchased from Sigma Chemical Co. (St. Louis, MO, USA). 3β-hydroxysteroid dehydrogenase (3β-HSD) was from Santa Cruz Biotechnologies (sc-30820, Santa Cruz, CA, USA). All other reagents were from Sigma or as indicated in the specified methods.

### Animals and experimental procedures

**Figure 1:**
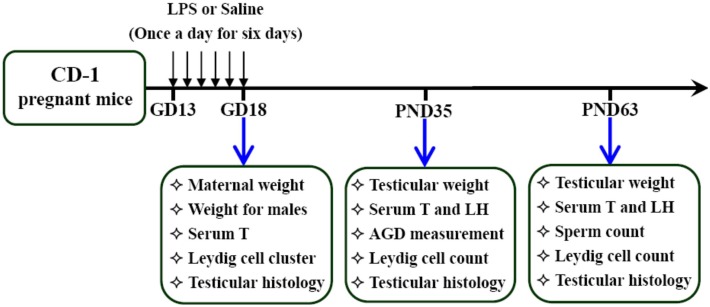
Schematic diagram summarizing timeline of experimental procedures in this study. The presence of a vaginal plug was designated as gestational day (GD) 0. Seventy-two pregnant mice were randomly divided into two groups. In LPS group, the pregnant mice were intraperitoneally injected with LPS (50 µg/kg) daily from gestational day (GD) 13 to GD18. The normal saline-treated pregnant mice served as controls. Six pregnant mice in each group were sacrificed. Fetal outcomes were evaluated. Within 24 h after birth, excess pups were removed, so that four males and four females were kept per dam. At postnatal day (PND) 21, pups were separated from the siblings and housed five to a cage. Testicular development, steroidogenesis and spermatogenesis were examined on PND 35 and 63, respectively.

Adult CD-1 male and female mice were purchased from the Center for Laboratory Animal in Anhui Province. All mice were fed ad libitum, and were housed in a room with controlled lighting (12-h light/12-h dark cycle) and temperature (22∼25°C). After acclimatization and quarantine, four female mice were mated with two males in a cage. The presence of a vaginal plug was designated as gestational day (GD) 0. Schematic diagram containing the timeline of experimental procedures in the current study was presented in Fig. 1. Seventy-two pregnant mice were randomly divided into two groups. In LPS group, the pregnant mice were intraperitoneally injected with LPS (50 µg/kg) daily from gestational day (GD) 13 to GD 17. The normal saline-treated pregnant mice served as controls. Our previous study showed no signs of maternal toxicity were observed in dams treated with LPS (50 ug/kg, i.p.) during late pregnancy [21]. In addition, maternal LPS (50 ug/kg, i.p.) exposure during late pregnancy did not result in preterm labor. A recent Meta-analysis showed humans might be exposed to this concentration during sepsis syndrome[22]. Therefore, in the present study, the pregnant mice were injected with LPS (50 ug/kg, i.p.) daily from gd 13 to gd 17. Twelve pregnant mice in each group were sacrificed by exsanguination after pentobarbital anesthesia (75 mg/kg, i.p.) treatments on GD 18. Fetuses were dissected, sexed and and weighed. Sera from male fetuses were collected and kept at –80°C for testosterone (T) measurement. Fetal testes were immersed in modified Davidson’s fluid (mDF) for 6 hr. Subsequently, testicular histology and immunohistochemistry (IHC) were detected.

The remaining pregnant mice were naturally delivered their fetuses. Within 24 h after birth, excess pups were removed, so that only ten pups were kept per dam. At postnatal day (PND) 21, pups were separated from the siblings and housed five to a cage. Anogenital distance (AGD) in males and females were evaluated at PND 26. At PND 35 and 63, twelve male mice from six litters in each group were euthanized by exsanguination after pentobarbital anesthesia (75 mg/kg, i.p.) treatments. Testes and prostates plus seminal vesicles were excised, dissected and weighed. Sera from male pups were collected and kept at –80°C for T and luteinizing hormone (LH) measurement. The left testis was immersed in mDF for 12–24 hr and detected for histology and immunohistochemistry. The number of spermatozoa in cauda epididymidis was counted at PND 63.

This study was approved by the Association of Laboratory Animal Sciences and the Center for Laboratory Animal Sciences at Anhui Medical University (Permit Number: 13-1182). All animal experimental procedures were performed in accordance with the guidelines for humane treatments established by the Association of Laboratory Animal Sciences and the Center for Laboratory Animal Sciences at Anhui Medical University.

### Hematoxylin & eosin staining

Testes were collected from male mice at GD18, PND 35 or 63. MDF-fixed testes were dehydrated and embedded in paraffin using standard procedures provided by Pathological Lab at Anhui Medical University. Paraffin-embedded tissues were serially cut. Sections of 5 µm were mounted onto glass slides, dewaxed with xylene, and rehydrated with graded ethanols. Six sections in each group were stained with hematoxylin and eosin (H&E) using standard procedures for morphological analyses. For adult testes, the seminiferous tubules were classified into three stage groups: stages I–VI, stages VII–VIII, and stages IX–XII. The method of classification was adopted to define the different stages of seminiferous tubules (Oakberg, 1956; Chiou et al., 2008). More than 150 tubules were classified for each section. The percent of three different stages of seminiferous tubules in total tubules was counted. Each experiment was repeated three times.

### Immunohistochemistry

Testicular Leydig cells were identified by immunostaining for 3β-HSD. Sections were mounted onto glass slides coated with poly-L-lysine, deparaffinized with xylene, and rehydrated with graded ethanol. Antigen retrieval was performed by microwave oven heating for 10–30 min in 0.01 M citrate buffer (pH 6.0). Slides were incubated for 10 min in 3% (vol/vol) hydrogen peroxide in PBS to block endogenous peroxidase activity and then washed in phosphate-buffered saline (PBS). After blocking in normal serum, the slides were overnight incubated with goat polyclonal antibody against 3β-HSD (diluted 1∶200 in special antibody dilutions; Santa Cruz, USA) at 4°C. After washing in PBS, slides were incubated with the appropriate secondary antibody conjugated to biotin at 37°C for 30 min. This was followed by incubation with streptavidin-peroxidase complex (Zhongshan Golden Bridge Biotechnology, Beijing) at 37°C for 30 min. Immunostaining was developed by application of 3,3′-diaminobenzidine (sigma), and slides were counterstained with hematoxylin, dehydrated, and mounted using neutral balsam. For fetal testes, quantification of Leydig cell (LC) clusters was performed using the public domain NIH Image J Program. Slides immunostained for 3β-HSD were of high contrast and low background to allow computer-assisted counting of clusters and determination of LC cluster area. LC clusters were then assigned to one of three groups: small clusters, accounting for ≤5% of the total LC cluster area per section; medium clusters, accounting for 5.1–14.9% of the total LC cluster area per section; large clusters, which individually accounted for ≥15% of the total LC cluster area per section. The cluster number and the proportion of each section occupied by all LC clusters were obtained as previously described [23]. For adult testes, the number of 3β-HSD-positive cells was counted in five randomly selected fields from each slide at a magnification of ×100.

### Radioimmunoassay (RIA)

Blood were collected from male offsprings at GD 18, PND 35, and PND 63. Serum was separated by centrifugation and stored at –80°C until assay for testosterone (T). ^125^I-based radioimmunoassay (RIA) kits were purchased from Beijing North Institute of Biological Technology (Beijing, China). T was measured according to the manufacturer’s protocol for serum samples. The concentration of serum T was expressed as ng/ml.

### Enzyme-linked immunosorbent assay (ELISA)

Mouse luteinizing hormone (LH) ELISA kits were purchased from USCN Life Science & Technology Co. (Wuhan, China). A competitive inhibition enzyme immunoassay was used to determine levels of LH in serum according to the manufacturer’s protocol. Briefly, fifty microliters of standard or sample were pipetted into wells precoated with specific antibody for LH. And then biotin-labeled LH (Detection Reagent A) was added and mixed immediately. The microplate was allowed to incubate for 1 hr at 37°C. After wells were rinsed four times in wash solution, an avidin conjugated horseradish peroxidase (HRP) was added to wells and incubated for 0.5 hr at 37°C. After wells were rinsed five times, a TMB substrate solution was added to wells and incubated for 20 min to yield a blue product. The reaction was stopped and the optical density was measured at 450 nm using a universal microplate reader (Bio-Tek Instruments, Inc., Winooski, VT, USA). For the kits used, the minimum detectable dose of LH was less than 145.5 pg/mL. All serum samples were diluted 1∶10 for LH in PBS before proceeding.

### Sperm count

The cauda epididymidis was dissected and immediately immersed into the pre-incubated F12 medium supplemented with 0.1% Bovine Serum Albumin (BSA). Spermatozoa were released by mincing the cauda epididymidis in the medium. The sperm suspensions were incubated for 5 minutes at 37°C. After incubation, sperm suspension was diluted with equal volume of sperm immobilization solution (100 mL distilled water, 1 mL 35% formalin and 5 g sodium bicarbonate) to immobilize the sperm. Sperm suspension was analyzed for sperm count according to WHO laboratory manual 4^th^ Edition. Twelve sperm samples from six litters each group were counted using an improved Neubauer haemocytometer. Assay for each sample was repeated two or three times.

### Statistical analyses

All data were given as means ± standard error of the mean (S.E.M.), and sample sizes are presented in figure legends. All statistical analyses were performed using SPSS 13.0 software. Differences between two groups were analyzed by means of a Student’s *t*-test. *P* value less than 0.05 was considered to be statistically significant.

## Results

### Effects of maternal LPS exposure during pregnancy on maternal weight gain and body weight of fetuses

**Figure 2:**
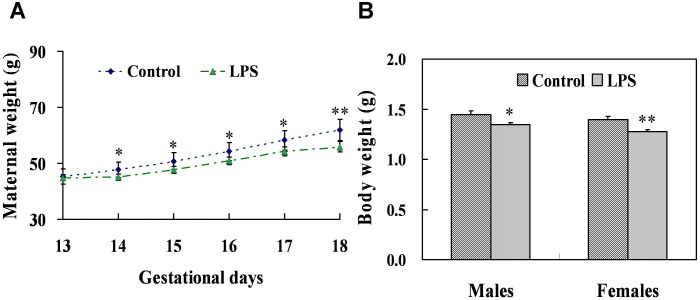
Body weights in maternal mice and its offspring. Maternal mice were injected with LPS (50 µg/kg, i.p.) daily from gestational day (GD) 13 to GD18. The dams were sacrificed on GD 18. (A) Maternal weights were recorded daily from GD13 to GD18. (B) Male and female fetuses were weighed separately on GD 18. Data were expressed as means ± SEM. *P<0.05, **P<0.01 as compared with controls.

To investigate the effects of maternal LPS exposure during pregnancy on maternal weight gain and body weight of fetuses, pregnant mice were injected with LPS (50 µg/kg, i.p.) daily from gestational day (GD) 13 to GD17. There were no signs of maternal toxicity in dams administered with LPS. As shown in Fig. 2A, maternal weight gain was significantly decreased in LPS-treated maternal mice as compared with the controls from GD14 to GD18. The effects of maternal LPS administration during pregnancy on weight of fetuses were presented in Fig. 2B. Results showed that fetal weight at GD18 were markedly reduced in males (*P* = 0.027) and females (*P* = 0.002) whose mothers were exposed to LPS during pregnancy. In addition, no significant difference was observed in male-to-female sex ratio (0.9±0.3 *vs* 1.0±0.6, *P* = 0.367) and the number of live fetuses per litter (11.0±3.0 *vs* 12.3±1.5, *P* = 0.065) between LPS group and the control group.

### Effects of maternal LPS exposure during pregnancy on histology and the distribution of leydig cells in fetal testes and T in fetal serum

**Figure 3:**
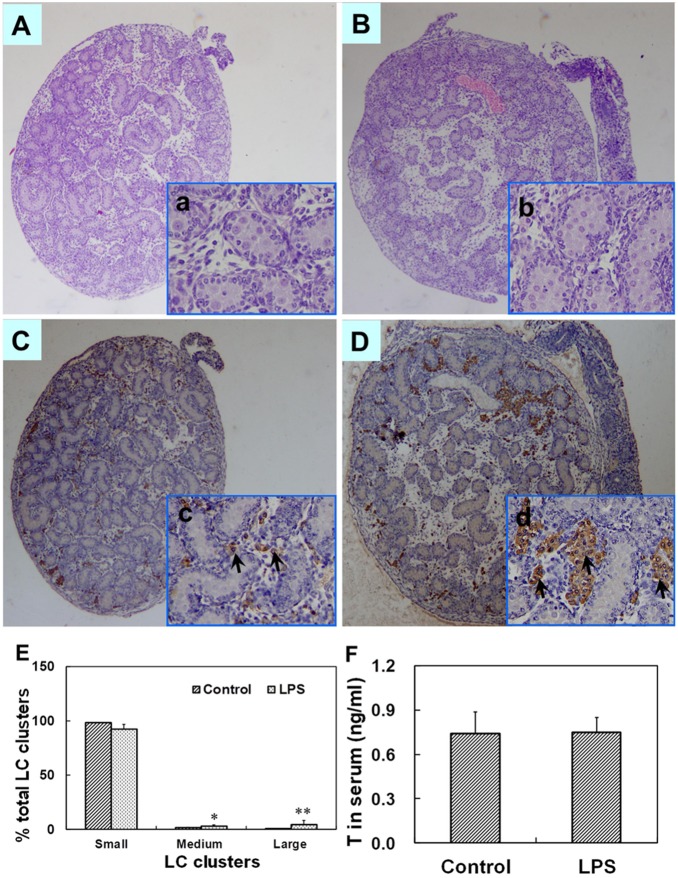
Testicular histology and serum T in male fetuses at GD 18. Maternal mice were injected with LPS (50 µg/kg, i.p.) daily from gestational day (GD) 13 to GD18. The dams were sacrificed on GD 18. Testes were collected from male fetuses at 6 h after the last LPS treatment. Testicular cross-sections from controls (A and a) and LPS-treated mice (B and b) were stained with H&E. (A and B) Magnification 50×; (a and b) Magnification 200×. Leydig cells in fetal testes from controls (C and c) and LPS-treated mice (D and d) were immunolocalized by staining with a polyclonal antibody against 3β-HSD. Arrows show 3β-HSD-positive cells. (C and D) Magnification 50×; (c and d) Magnification 200×. (E) Distribution of Leydig cell (LC) clusters in fetal testes was analyzed. Small clusters accounting for ≤5% of total LC cluster area per testis, medium clusters for 5.1–14.9% and large clusters for ≥15% of total LC cluster area per testis. (F) Serum T in male fetuses was measured by RIA. Data were expressed as means ± SEM. *P<0.05, **P<0.01 as compared with controls.

The effects of maternal LPS exposure during pregnancy on testicular histology of fetuses were presented as Figs. 3A–D. Testicular H&E staining showed that no abnormal morphological changes in fetal testes whose mothers were treated with LPS. Testicular Leydig cells in fetal mice were identified by immunostaining using 3β-HSD. The effects of maternal LPS exposure during pregnancy on the distribution of testicular Leydig cells in fetuses were presented as Figs. 3C–E. Maternal LPS exposure slightly reduced small LC (Leydig cell) clusters as compared with the controls. In addition, a significant increase in medium and large LC clusters was observed in male fetuses whose mothers were exposed to LPS during pregnancy. To investigate the effects maternal LPS exposure during pregnancy on T synthesis in fetal testes, serum T was measured by RIA. As shown in Fig. 3F, no significant difference in the level of serum T was observed between two groups (*P* = 0.473).

### Effects of prenatal LPS exposure on weight of gonads and testicular histology at PND 35

**Figure 4:**
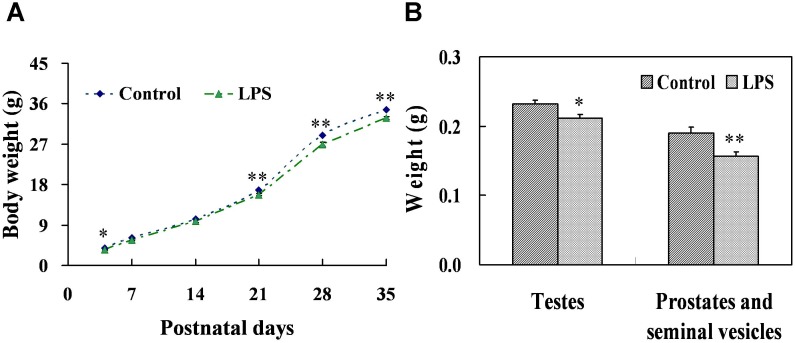
Body weight and gonads weight in male mice at PND 35. Maternal mice were injected with LPS (50 µg/kg, i.p.) daily from gestational day (GD) 13 to GD18. Within 24 h after birth, excess pups were removed, so that four males and four females were kept per dam. At postnatal day (PND) 21, pups were separated from the siblings and housed five to a cage. Some males each group were sacrificed on PND 35. Testes, prostates plus seminal vesicles were collected from male offsprings. (A) Body weight in male offspring was recorded from PND 4, 7, 14, 21, 28 and 35. (B) Testes and prostates plus seminal vesicles were weighed separately on PND 35. Data were expressed as means ± SEM of twelve samples from six litters. *P<0.05, **P<0.01 as compared with controls.

**Figure 5:**
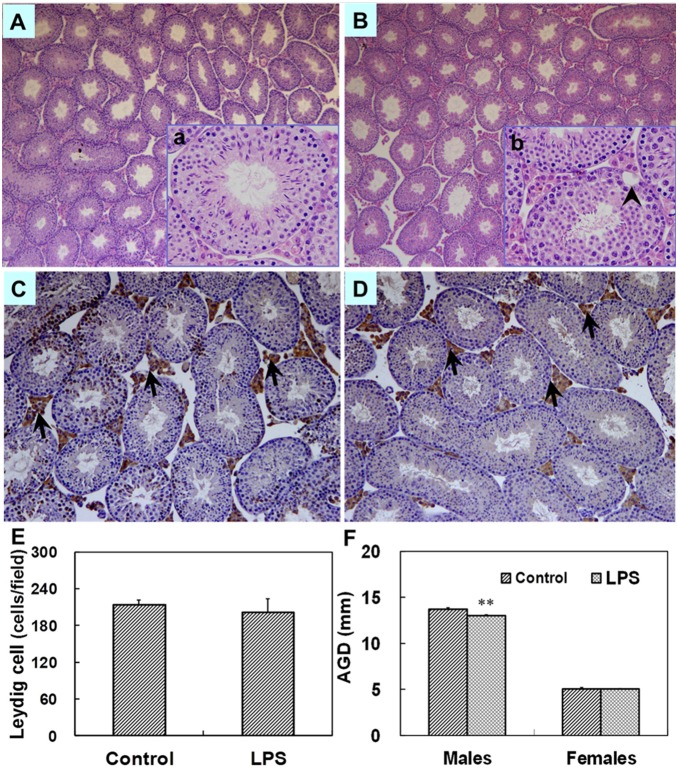
Testicular histology and AGD in male mice during puberty. Maternal mice were injected with LPS (50 µg/kg, i.p.) daily from gestational day (GD) 13 to GD18. Within 24 h after birth, excess pups were removed, so that four males and four females were kept per dam. At postnatal day (PND) 21, pups were separated from the siblings and housed five to a cage. Some males each group were sacrificed on PND 35. Testes were collected from male offsprings. Testicular cross-sections from controls (A and a) and LPS-treated mice (B and b) were stained with H&E. (A and B) Magnification 50×; (a and b) Magnification 200×. Arrowhead shows a large vacuole. Leydig cells in testes from controls (C) and LPS-treated mice (D) were immunolocalized by staining with a polyclonal antibody against 3β-HSD at magnification 100×. Arrows show 3β-HSD-positive cells. (E) The number of testicular Leydig cells was counted. Five fields were randomly selected from each section at magnification 100×. (F) Anogenital distance (AGD) in males and females was examined on PND 26. Data were expressed as means ± SEM. **P<0.01 as compared with controls.

As shown in Fig. 4A, prenatal LPS exposure obviously delayed body weight gain of male offsprings at postnatal day (PND) 4, 21, 28 and 35. The effects of prenatal LPS exposure on weight of testes, prostates and seminal vesicles at PND 35 were presented in Fig. 4B. Results showed that the weight of testes (*P* = 0.031), prostates and seminal vesicles (*P* = 0.004) at PND 35 were significantly decreased in pups whose mothers were exposed to LPS. Testicular histology at PND 35 was also assessed. H&E staining demonstrated that no morphogical changes except several large vacuoles were observed in males at PND35 (Figs. 5A–B).

### Effects of prenatal LPS exposure on T and LH in serum, the number of testicular leydig cells at PND 35 and AGD at PND 26

**Figure 6:**
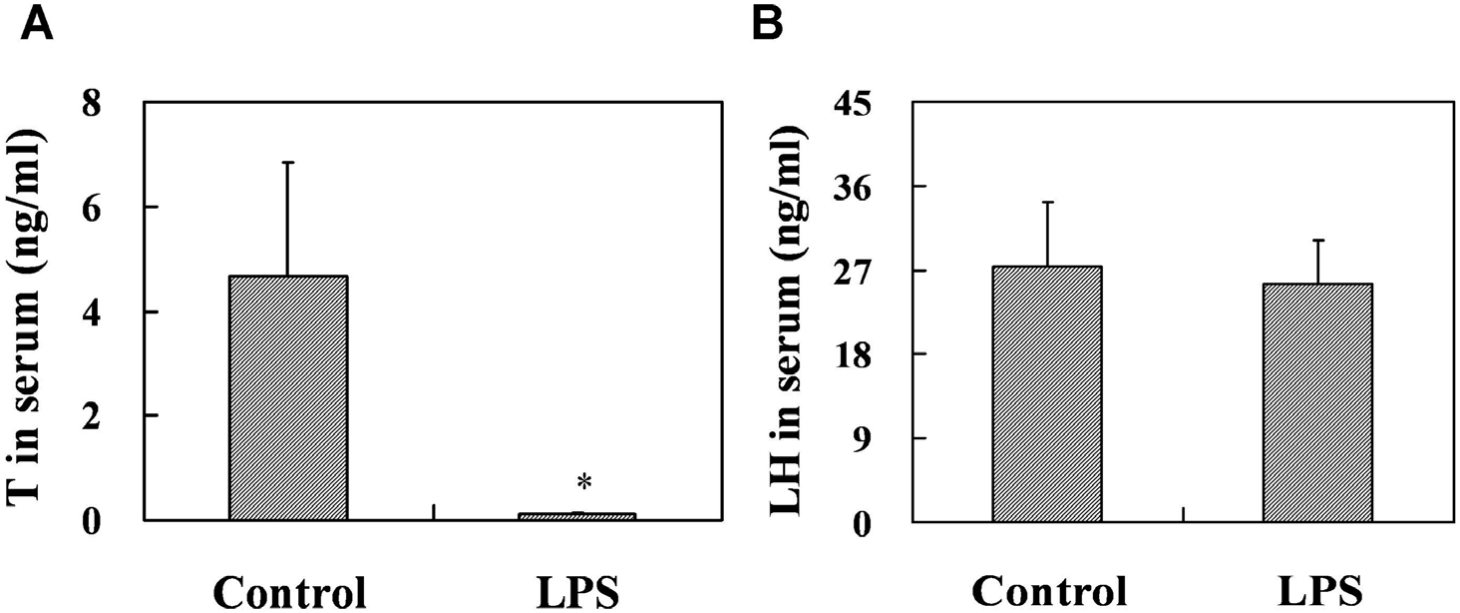
Serum T and LH in male mice at PND 35. Maternal mice were injected with LPS (50 µg/kg, i.p.) daily from gestational day (GD) 13 to GD18. Within 24 h after birth, excess pups were removed, so that four males and four females were kept per dam. At postnatal day (PND) 21, pups were separated from the siblings and housed five to a cage. Some males each group were sacrificed on PND 35. Sera were collected from male offsprings. (A) Serum T in males was measured by RIA. (B) Serum LH in males was measured by ELISA. Data were expressed as means ± SEM of twelve samples from six litters. *P<0.05 as compared with controls.

To explore the effects of prenatal LPS exposure on T and LH in serum from PND 35 male mice, serum T and LH were measured by RIA and ELISA, respectively. Prenatal LPS exposure significantly decreased the level of serum T at PND 35 (*P* = 0.027), whereas no obvious difference in the level of serum LH (*P* = 0.440) was observed in male mice at PND 35 (Figs. 6A and B). Leydig cells in mouse testes were identified by immunostaining for 3β-HSD. As described in Figs. 5C–E, prenatal LPS exposure had no effects on the number of testicular leydig cells at PND 35 (*P* = 0.307). The effects of prenatal LPS exposure on anogenital distance (AGD) at PND26 were presented in Fig. 5F. Results showed that prenatal LPS treatment markedly shortened AGD in male mice at PND 26 (*P* = 0.0003), whereas no significant difference in AGD was observed in females between two groups (*P* = 0.320).

### Effects of prenatal LPS exposure on weight of gonads, testicular histology and the number of sperm at PND 63

**Figure 7:**
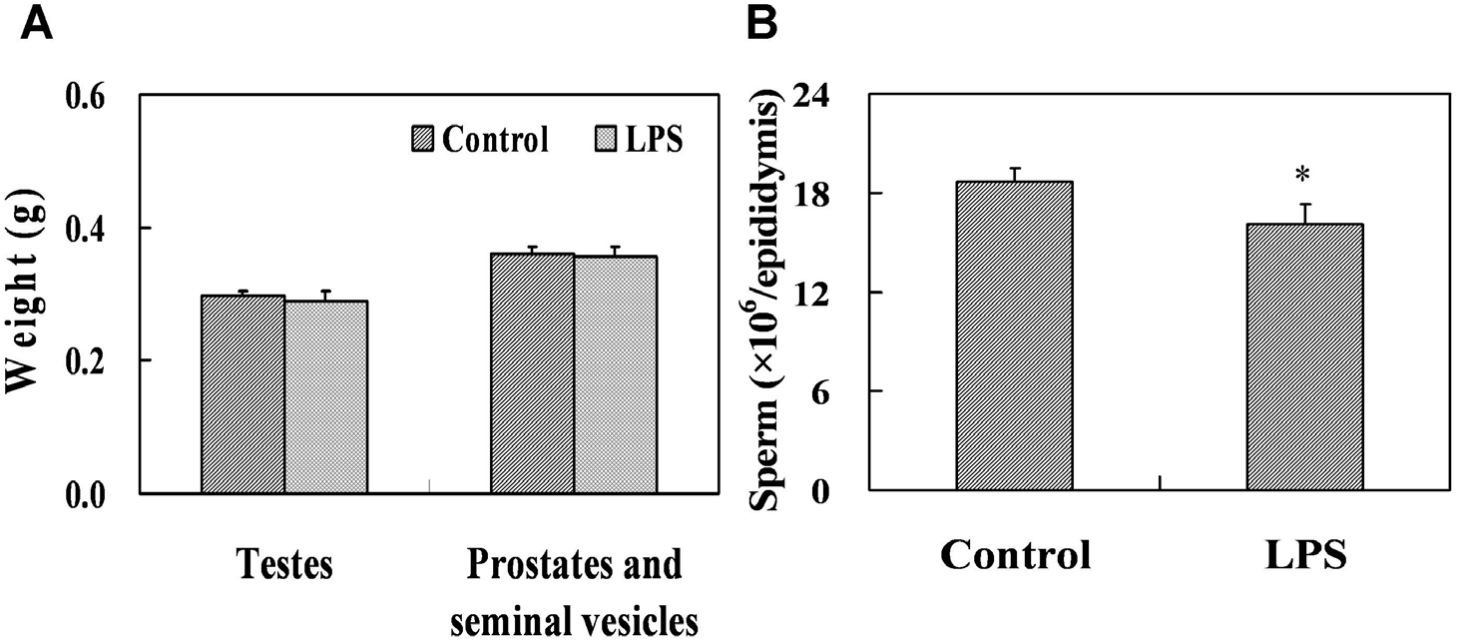
Gonads weight and the number of sperm in male mice at PND 63. Maternal mice were injected with LPS (50 µg/kg, i.p.) daily from gestational day (GD) 13 to GD18. Within 24 h after birth, excess pups were removed, so that four males and four females were kept per dam. At postnatal day (PND) 21, pups were separated from the siblings and housed five to a cage. Some males each group were sacrificed on PND 63. Testes, prostates plus seminal vesicles were collected from male offsprings. (A) Testes and prostates plus seminal vesicles were weighed separately on PND 63. (B) The number of sperm per epididymis was counted on PND 63. Data were expressed as means ± SEM of twelve samples from six litters. *P<0.05 as compared with controls.

**Figure 8:**
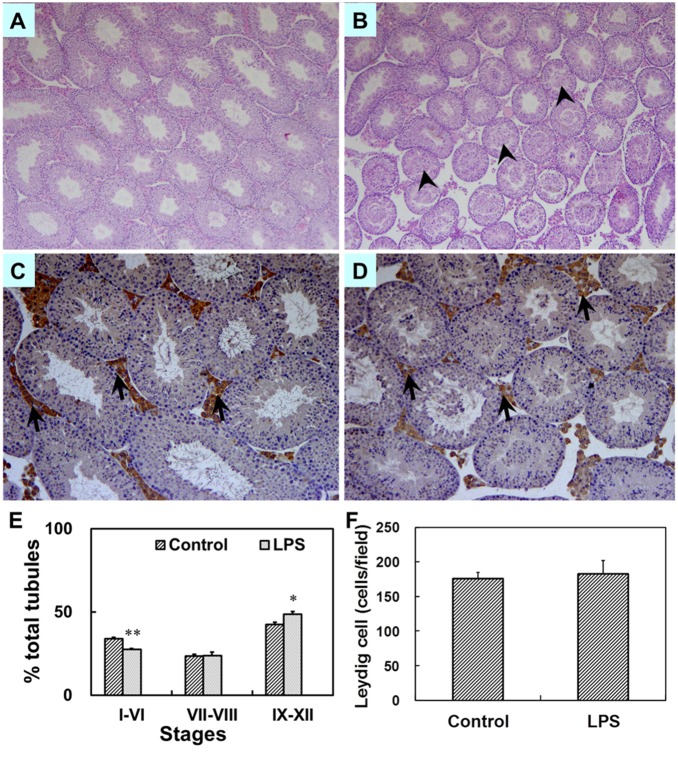
Testicular histology in male mice at PND 63. Maternal mice were injected with LPS (50 µg/kg, i.p.) daily from gestational day (GD) 13 to GD18. Within 24 h after birth, excess pups were removed, so that four males and four females were kept per dam. At postnatal day (PND) 21, pups were separated from the siblings and housed five to a cage. Some males each group were sacrificed on PND 63. Testes were collected from male offsprings. Testicular cross-sections from controls (A) and LPS-treated mice (B) were stained with H&E at magnification 50×. Arrowheads show massive sloughed germ cells in the lumen of tubules. Leydig cells in testes from controls (C) and LPS-treated mice (D) were immunolocalized by staining with a polyclonal antibody against 3β-HSD at magnification 100×. Arrows show 3β-HSD-positive cells. (E) The percent of three different stages of seminiferous tubules in total tubules was counted. Testicular cross-sections were stained by H&E. The cycle of seminiferous tubules was classified into three stage groups: stages I–VI, VII–VIII, IX–XII. Data were expressed as means ± SEM of six sections from six litters. More than 150 tubules per slide were observed. *P<0.05, **P<0.01 as compared with controls. (F) The number of testicular Leydig cells per field was counted. Five fields were randomly selected from each section at magnification 100×. Data were expressed as means ± SEM of six sections from six litters.

The effects of prenatal LPS exposure on weight of testes, prostates and seminal vesicles at PND 63 were presented in Fig. 7A. Results showed that no significant difference in the weight of testes, prostates and seminal vesicles at PND 63 was occurred between the two groups. However, prenatal LPS administration markedly reduced the number of spermatozoa in the cauda epididymidis of mice at PND63 (Fig. 7B, *P* = 0.044). Testicular histology at PND 63 was also assessed by H&E staining. As reported in Figs. 8A and B, prenatal LPS exposure caused massive sloughing of germ cells in the majority of seminiferous tubules in mouse testes at adulthood. Spermatocytes and spermatids consisted of the sloughed germ cell in the lumen of seminiferous tubules. Fig. 8E showed the percent of the cycle of seminiferous tubules in different stages. In control males, 33.9% (88.8±4.4), 23.6% (61.5±2.8) and 42.5% (112.8±10.5) of the seminiferous tubules per section were in stages I–VI, VII–VIII and IX–XII, respectively. In LPS-treated males, 27.6% (79.8±4.1), 23.9% (68.8±6.0) and 48.6% (142.2±11.2) of the seminiferous tubules were in stages I–VI, VII–VIII and IX–XII, respectively. As shown in Fig. 8E, prenatal LPS exposure significantly diminished the percent of seminiferous tubules in stages I–VI (*P* = 0.00003), whereas the percent of seminiferous tubules in stages IX–XII were markedly risen in LPS-treated males (*P* = 0.010).

### Effects of prenatal LPS exposure on T and LH in serum, the number of testicular leydig cells at PND 63

**Figure 9:**
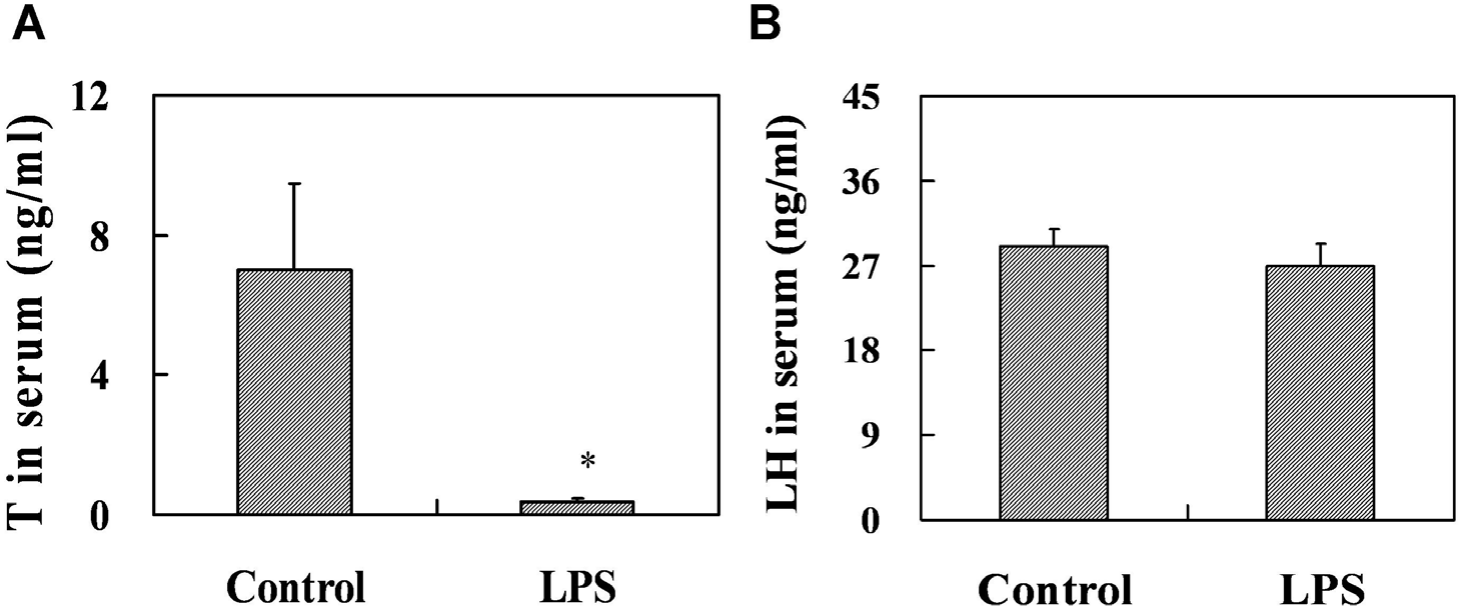
Serum T and LH in male mice at PND 63. Maternal mice were injected with LPS (50 µg/kg, i.p.) daily from gestational day (GD) 13 to GD18. Within 24 h after birth, excess pups were removed, so that four males and four females were kept per dam. At postnatal day (PND) 21, pups were separated from the siblings and housed five to a cage. Some males each group were sacrificed on PND 63. Sera were collected from male offsprings. (A) Serum T in males was measured by RIA. (B) Serum LH in males was measured by ELISA. Data were expressed as means ± SEM of twelve samples from six litters. *P<0.05 as compared with controls.

The effects of prenatal LPS exposure on T and LH in serum from PND 63 males were presented in Figs. 9A and B. Results showed that prenatal LPS exposure significantly decreased the level of serum T at PND 63 (*P* = 0.016), whereas no significant difference in the level of serum LH was observed in male mice at PND 63 (*P* = 0.148). To investigate the effects of prenatal LPS exposure on the number of testicular leydig cells, we detected 3β-HSD-positive leydig cells by immunohistochemistry. As shown in Figs. 8C, D and F, prenatal LPS exposure had no effects on the number of testicular leydig cells at PND 63 (*P* = 0.391).

## Discussion

LPS is associated with transitive or permanent male infertility. Several studies demonstrated that adult LPS exposure impaired testicular histology and spermatogenic function in rats [24]–[26]. A recent study showed that neonatal LPS exposure disrupted puberty onset and sexual performance, reduced gonocyte presence per tubule, and increased the disorganization of seminiferous epithelium in rats [27]. In the present study, we explored the effects of maternal LPS exposure during pregnancy on testicular development and spermatogenesis in male offspring. Our results showed that the weight of testes at PND 35 and the number of sperm at PND63 were significantly decreased in pups whose mothers were exposed to LPS during pregnancy. Furthermore, maternal LPS exposure during pregnancy led to massive sloughing of germ cells in seminiferous tubules, diminished the percent of seminiferous tubules in stages I–VI, and increased the percent of seminiferous tubules in stages IX–XII in mouse testes at adulthood. These results indicate that maternal LPS exposure during pregnancy permanently impairs testicular development and spermatogenesis in male offspring.

T is essential to maintain normal testicular development, spermatogenesis, and male fertility [28], [29]. T actions are mediated by androgen receptor (AR) in testes. Specific knock out of AR in Sertoli or Leydig cells caused male infertility with spermatogenic arrest and hypotestosteronemia [30]–[32]. Several earlier studies demonstrated that acute LPS challenge caused a marked decrease in serum T level in adult male mice and rats [33], [34]. In the current study, we investigated whether maternal LPS exposure decreased T production in male offspring. Results showed that serum T level was significantly diminished in male mice whose mothers were exposed to LPS during pregnancy. Correspondingly, maternal LPS exposure during pregnancy markedly shortened anogenital distance (AGD), an index of altered androgen action, in male mice at PND 26. Together, we infer that a marked decrease in T production may be responsible for testicular histological injury and impaired spermatogenesis in LPS-treated males.

In males, T is primarily synthetized in testicular Leydig cells. A variety of studies demonstrate that the number and distribution of testicular Leydig cells influences intra-testicular T production [35]–[37]. Several earlier studies found that abnormal Leydig cell aggregation in fetal testes was linked with reduced concentration of testicular T in rats exposed to di(n-butyl) phthalate [23], [37]. In this work, we investigated the effects of maternal LPS exposure during pregnancy on the number and distribution of Leydig cells in male offspring. Results showed that maternal LPS exposure during pregnancy obviously increased the medium and large LC clusters in male fetal testes, whereas the number of testicular Leydig cells at puberty or adulthood wasn’t altered in LPS-treated males. Therefore, the present results suggest that maternal LPS exposure during pregnancy disrupts testicular T synthesis, at least partially, via altering Leydig cellular distribution.

It has been known that T synthesis in testicular Leydig cells is mainly under the control of the pituitary gonadotropin luteinizing hormone (LH). Previous studies demonstrated that adult LPS challenge obviously reduced serum LH level, and subsequently inhibited intra-testicular T synthesis in male rats [38], [39]. Recently, several studies found that no significant alterations in serum LH concentration were observed in adolescent or adult males following neonatal LPS treatment [27], [39]. In the present study, we explored the effects of maternal LPS exposure during pregnancy on circulating LH and T level in male offspring. Our results showed that, although a decrease of serum T level was occurred in LPS-treated males at PND 35 and PND 63, no significant difference in serum LH concentration was observed in males at PND 35 and PND 63. Thus, we can rule out the possibility that maternal LPS exposure during pregnancy suppresses testicular T synthesis via impairing hypothalamic-pituitary- gonadal (HPG) axis.

Recently, a decline in semen quality was observed in many young healthy men worldwide [40]. Apart from genetic factors, environmental factors also contributed to impairments of semen quality, such as sperm count, morphology, motility and viability. A variety of studies indicated that there was a strong correlation between impaired semen quality and acute exposure to heavy metals, pesticides, industrial chemicals, endocrine disruptors, chemotherapy, radiation, genitor-urinary tract infection, unhealthy habits [41], [42]. In the current study, we for the first time report that maternal LPS exposure during pregnancy leads to a decrease in the number of sperm in male offspring. Thus, our results will provide a theoretic basis for clarifying the cause of male infertility and making its prevetion and control measures.

In summary, we investigated the effects of maternal LPS exposure during pregnancy on testicular development, steroidogenesis and spermatogenesis in male offspring. Results showed that maternal LPS exposure during pregnancy led to a significant decrease in body weight and abnormal Leydig cell aggregations in male fetuses on GD18, a reduction in the weight of testes, prostates and seminal vesicles in male offspring at puberty, a decline of serum testosterone level in male offspring at puberty and adulthood, a decrease in sperm count and massive sloughing of germ cells in seminiferous tubules of male offspring at adulthood. Taken together, these results suggest that maternal LPS exposure during pregnancy disrupts T production. The decreased T synthesis might be associated with LPS-induced spermatogenesis impairments in male offspring.

## Supporting Information

Checklist S1 **ARRIVE Guidelines Checklist.**
(DOC)
